# PACAP and VIP Mitigate Rotenone-Induced Inflammation in BV-2 Microglial Cells

**DOI:** 10.1007/s12031-022-01968-1

**Published:** 2022-02-24

**Authors:** Sarah Thomas Broome, Giuseppe Musumeci, Alessandro Castorina

**Affiliations:** 1 Laboratory of Cellular and Molecular Neuroscience (LCMN), School of Life Sciences, Faculty of Science, University of Technology Sydney, PO Box 123, Broadway, NSW 2007 Australia; 2 Section of Human Anatomy, Histology and Movement Science, Department of Biomedical and Biotechnological Sciences, University of Catania, via S. Sofia, 87, 95123 Catania, Italy

**Keywords:** Pituitary adenylate cyclase-activating peptide (PACAP), Vasoactive intestinal peptide (VIP), Rotenone, Microglia, Neuroinflammation

## Abstract

Rotenone is a commercial pesticide commonly used to model Parkinson’s disease (PD) due to its ability to induce dopaminergic degeneration. Studies have confirmed that rotenone causes microglial activation, which seems to contribute to the toxic effects seen in rodent models. Pituitary adenylate cyclase–activating polypeptide (PACAP) and vasoactive intestinal peptide (VIP) are two structurally related neuropeptides that have robust neuroprotective and anti-inflammatory properties. However, their ability to regulate microglial activity in response to rotenone is not fully understood. Using rotenone as an inflammatory stimulus, we tested whether PACAP or VIP could mitigate microglial activation in BV2 microglial cells. Rotenone dose-dependently reduced cell viability and the percentage of apoptotic cells. It also increased the release of nitric oxide (NO) in culture media and the expression of microglial activation markers and pro-inflammatory markers, including CD11b, MMP-9 and IL-6, and heightened the endogenous levels of PACAP and its preferring receptor PAC1. Co-treatment with PACAP or VIP prevented rotenone-induced increase of NO, CD11b, MMP-9 and IL-6. These results indicate that both PACAP and VIP are able to prevent the pro-inflammatory effects of rotenone in BV2 cells, supporting the idea that these molecules can have therapeutic value in slowing down PD progression.

**Graphical Abstract:**

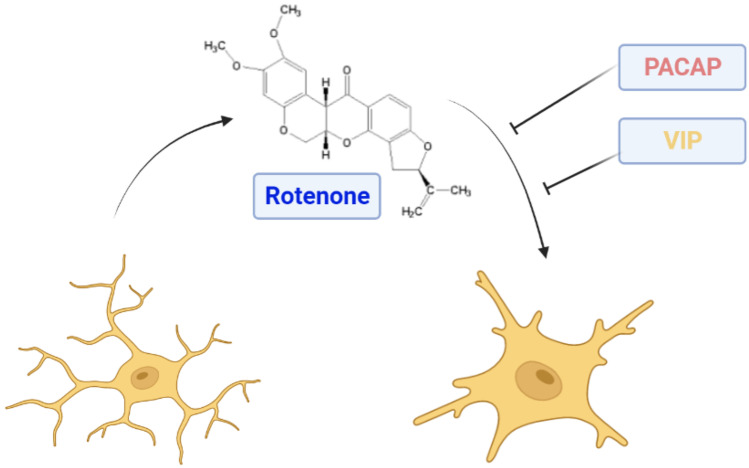

## Introduction

Parkinson’s disease (PD) is a progressive neurodegenerative disease that is characterised by the degeneration of dopaminergic neurons in the *substantia nigra pars compacta*, resulting in a deficit of dopamine in the striatum (Glass et al. 2010; Schapira et al. 2017). The consequent nigro-striatal depletion of dopamine is responsible for a myriad of symptoms, including not only bradykinesia, postural instability and resting tremor, but also cognitive and mood disorders and autonomic dysfunctions (Braak et al. 2004; Sveinbjornsdottir 2016). Unfortunately, the cause of PD remains unknown; however, a range of risk factors have been identified, including genetics, age and environmental exposure to toxicants (Cacabelos 2017).

Rotenone is a commercial pesticide and piscicide that is used to model PD due to its ability to selectively damage dopaminergic neurons (Alam and Schmidt 2002; Radad et al. 2019). Rotenone has become popular as it can mimic several key pathological features of the disease, including dopaminergic degeneration, mitochondrial dysfunction, α-synuclein aggregation and neuroinflammation (Betarbet et al. 2000; Miyazaki et al. 2020).

Neuroinflammation is considered one of the main pathological mechanisms of PD, with inflammation alone being able to induce dopaminergic cell death (Castano et al. 1998; Hunter et al. 2007). Microglia are the predominant cell types regulating neuroinflammation in the central nervous system (CNS), and these cells produce most of the pro-inflammatory and neurotoxic mediators (Cherry et al. 2014; Janda et al. 2018). Factors that can mitigate microglial activation might display neuroprotective effects in PD (Gupta et al. 2018; Thomas Broome et al. 2021a). For example, it has been shown that rotenone induces microglial activation in mice and suppression of rotenone-induced microglial activation renders mice more resistant to degeneration in models of PD (Jing et al. 2021). Furthermore, studies have shown that rotenone-induced microglial activation contributes to neurodegeneration (Sharma et al. 2020) and cognitive impairment (Zhang et al. 2021). Notably, it has been shown that rotenone caused microglial activation before anatomical evidence of dopaminergic degeneration was observed in a rodent model of PD (Sherer et al. 2003).

Pituitary adenylate cyclase–activating polypeptide (PACAP) and vasoactive intestinal peptide (VIP) are neuropeptides endowed with a range of neuroprotective and immunomodulatory functions (Castorina et al. 2010; Dejda et al. 2005; Giunta et al. 2010; Waschek 2013). Both PACAP and VIP have been shown to exert neuroprotective effects in dopamine-rich regions of the CNS (Delgado et al. 2002; Hirabayashi et al. 2018). Additionally, PACAP protects extra-nigral regions, including the hippocampus, prefrontal cortex and amygdala from inflammatory insults and enhances aversive learning (Mandwie et al. 2021; Marzagalli et al. 2016). Furthermore, both peptides demonstrated to possess potent anti-inflammatory activities in the CNS and periphery (Abad and Waschek 2011; Carniglia et al. 2017; Gonzalez-Rey et al. 2010; Masmoudi-Kouki et al. 2007). At the cellular level, PACAP and VIP have shown to be able to reduce the polarization of microglia driven by several inflammatory mimetics (Brown et al. 2014; Karunia et al. 2021).

In the context of PD, the administration of either peptide has shown to be neuroprotective. For example, PACAP administration protected against MPTP dopaminergic degeneration (Lamine et al. 2016) and VIP treatment modulated inflammation to prevent MPTP and 6-OHDA-induced degeneration (Korkmaz et al. 2012; Olson et al. 2015). Of note, PACAP has shown to be neuroprotective against rotenone-induced toxicity in both PC12 cells and snail models of PD (Maasz et al. 2017; Wang et al. 2005). However, it is still unclear if these peptides can also prevent inflammation in microglial cells exposed to the PD-mimetic rotenone.

In the present work, we tested this hypothesis using morphological, biochemical and molecular analyses. Results demonstrated that these peptides prevented microglial polarization, nitric oxide release as well as the induction of a range of pro-inflammatory markers in response to rotenone-induced inflammation, hence supporting the theory that PACAP and VIP are effective anti-inflammatory agents.

## Materials and Methods

### Cell Cultures

Mouse microglial BV-2 cells were grown in full growth media containing Dulbecco’s modified Eagle’s medium nutrient mixture F-12 HAM (1:1 *vol*/*vol* DMEM/F12) (Sigma-Aldrich, Castle Hill, NSW, Australia), 10% heat-inactivated foetal bovine serum (FBS, Scientifix Australia, Clayton, VIC, Australia) and 1% penicillin/streptomycin solution (Sigma-Aldrich, Castle Hill, NSW, Australia) and were stored in an incubator with humidified air containing 5% CO_2_ at a temperature of 37 °C. Cells were serum-starved in 1% FBS 24 prior to treatment. When cells reached 80–85% confluence, they were treated as indicated for 24 h.

### Cell Viability (MTT Assay)

To assess cell viability, we used the cell proliferation kit I (Sigma-Aldrich, NSW, Australia). Cells were treated with increasing doses of rotenone (0.0001–1 µM) and/or PACAP or VIP (0.0001–1 µM, respectively) for 24 h. Supernatant was removed and used for Griess reagent assay (please see below). Ten microlitres of MTT labelling reagent was added to each well of a 96-well plate for 4 h. Cells were incubated overnight in 100 µL of solubilization solution. Absorbance was measured at 656 nm in the TECAN infinite M1000-PRO ELISA reader (Thermo Fisher Scientific, Victoria, Australia). Optical density values were recorded and reported as a percentage of control.

### Relative Nitric Oxide Measurements (Griess Reagent Assay)

Cells were treated with the indicated treatments for 24 h before supernatant was transferred to a new 96-well plate. One hundred microlitres of Griess reagent (Sigma-Aldrich, NSW, Australia) were added to each well and kept for 15 min on a slow oscillation at room temperature. Absorbance was measured at 540 nm using the TECAN infinite M1000-PRO ELISA reader (Thermo Fisher Scientific, Victoria, Australia). Optical density values were recorded and reported as a percentage of control.

### Morphological Analysis

**Fig. 1:**
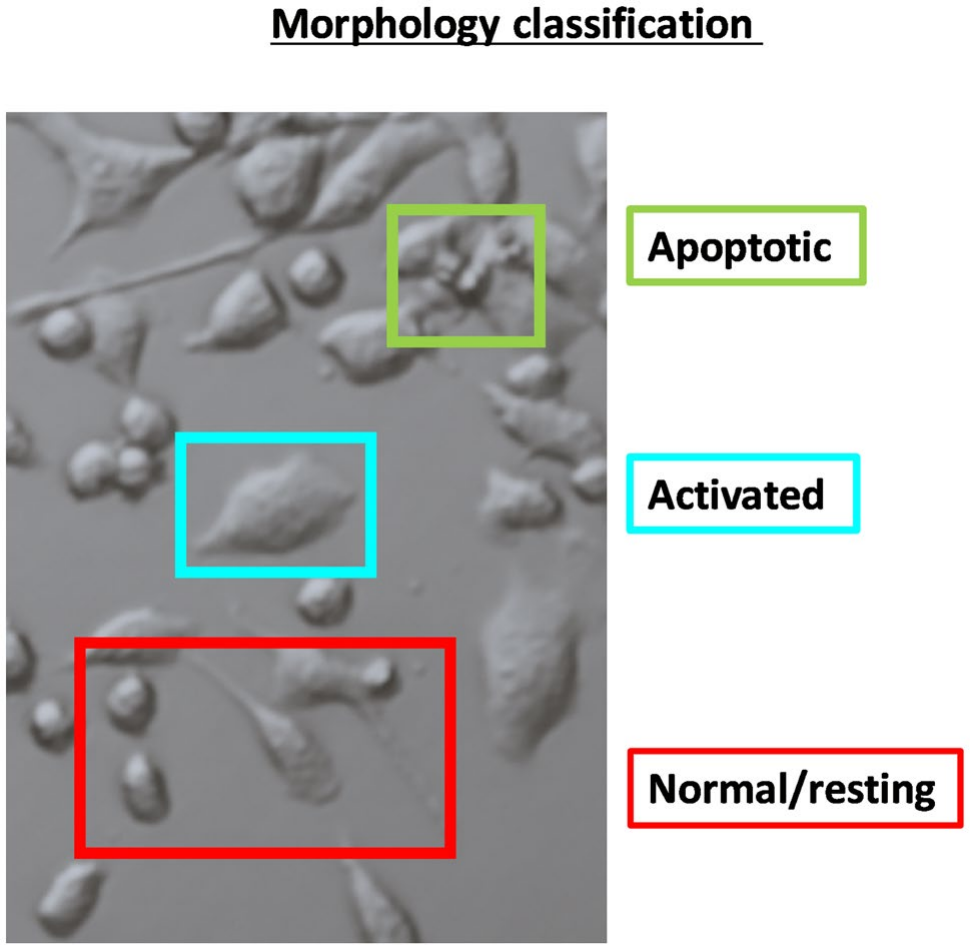
Morphological classification of microglial phenotypes. BV2 microglial cells were grossly classified into three categories: (1) normal/resting based on their rounded/spindle-like/nultipolar appearance with or without processes (red), (2) activated based on flattened and swollen appearance with no processes (blue), or (3) apoptotic based on shrinkage and fragmentation (green)

BV2 cells were seeded in 96-well plates for cell viability analysis and treated with the indicated treatments for 24 h. Images were then captured using a Nikon Eclipse TS2 inverted microscope (using the embossing filter settings) and subjected to morphological assessment (magnification × 20). Images were de-identified for analysis. Gross morphology was assessed based on the following three categories: (1) resting cells (rounded/spindle-like/multipolar cells, with or without thin processes), (2) activated (flattened cell body, swollen somata and/or thick retracted processes) or (3) apoptotic (shrinked and/or fragmented cells [apoptotic bodies]), as summarised in Fig. 1. Analyses of microscope images were performed using Fiji ImageJ, where cells were labelled into individual categories, counted and expressed as a percentage of total cells.

### RT-qPCR

Total RNA was extracted using TRI reagent (Sigma-Aldrich, NSW, Australia) and precipitated with ice-cold 2-propanol (Sigma-Aldrich). Pellets were washed twice with 75% ethanol and air-dried. RNA concentrations were calculated using NanoDrop™ 2000 (Thermo Fisher Scientific). A total of 1 µg of total RNA was used to synthesised cDNA using the Tetro cDNA Synthesis Kit (Bioline, Australia). Real-time qPCR analyses were performed as previously reported (Leggio et al. 2015; Thomas Broome et al. 2021a). Briefly, for each gene of interest, 3 µL cDNA, 0.4 µL Milli-Q water, 5 µL iTaq Universal SYBR Green Master Mix (Bio-Rad) and 0.8 µL corresponding forward and reverse primers (5 µM) for the gene of interest (detailed in Table 1) were combined and detected using the CFX96 Touch TM Real-Time PCR Detection System (Bio-Rad, NSW, Australia). Instrument settings were as follows: (1) 95 °C for 2 min, (2) 60 °C for 10 s, (3) 72 °C for 10 s, (4) plate read, (5) repeat step 2 to 4 for 45 cycles. For the melting curve analyses, settings were (1) 65 °C for 35 s, (2) plate read, and (3) repeat step 1–2 for 60 times). To examine changes in gene expression, we analysed the mean fold change values of each sample, calculated using the ΔΔCt method, as previously described by Schmittgen and Livak (2008). PCR product specificity was evaluated by melting curve analysis, with each gene showing a single peak (data not shown).

**Table 1:** List of primer sets used in real-time qPCR analysis. Forward and reverse primers were selected from the 5′ and 3′ region of each gene mRNA. The expected length of each amplicon is indicated in the right column

**Accession no**	**Gene**	**Primer sequence (5′-3′)**	**Length (bp)**
NM_000600.4	Interleukin-6 (IL-6)	Fwd TGACCCAACCACAAATGCCA Rev ATTTGCCGAAGAGCCCTCAG	135
NM_001082960.1	CD11b	Fwd GAGCAGGGGTCATTCGCTAC Rev GCTGGCTTAGATGCGATGGT	94
NM_013599.4	Matrix metallopeptidase 9 (MMP-9)	Fwd ATCATAGAGGAAGCCCATTACAG Rev TTTGACGTCCAGAGAAGAAGAAA	129
NM_010927.4	Nitric oxide synthetase 2 (NOS2)	Fwd TACCAAAGTGACCTGAAAGAGG Rev TCATCTTGTATTGTTGGGCTGA	89
NM_007482.3	Arginase-1 (Arg1)	Fwd ACAAGACAGGGCTCCTTTCAG Rev TTAAAGCCACTGCCGTGTTC	105
NM_010548.2	Interleukin-10 (IL-10)	Fwd GCATGGCCCAGAAATCAAGG Rev GAGAAATCGATGACAGCGCC	91
NM_016989.2	Pituitary adenylate-cyclase-activating polypeptide (PACAP)	Fwd AGGCTTACGATCAGGACGGA Rev CTCCTGTCGGCTGGGTAGTA	121
NM_053991.1	Vasoactive intestinal peptide (VIP)	Fwd CCTGGCGATCCTGACACTCT Rev CTGCAGCCTGTCATCCAACC	100
NM_007407.3	PAC1 receptor	Fwd CAGTCCCCAGACATGGGAGGCA Rev AGCGGGCCAGCCGTAGAGTA	139
NM_011703.4	VPAC1 receptor	Fwd TCAATGGCGAGGTGCAGGCAG Rev TGTGTGCTGCACGAGACGCC	127
NM_009511.2	VPAC2 receptor	Fwd GCGTCGGTGGTGCTGACCTG Rev ACACCGCTGCAGGCTCTCTGAT	155
NM_213557.1	18S ribosomal subunit (s18)	Fwd GGCGGAAAATAGCCTTCGCT Rev AGCCCTCTTGGTGAGGTCAA	101

### Western Blots

Protein was extracted using a radioimmunoprecipitation assay (RIPA) buffer containing protease inhibitors to preserve protein integrity (cOmplete, Mini, EDTA-free Protease Inhibitor Cocktail, Sigma-Aldrich, Castle Hill, NSW, Australia) as previously reported (Thomas Broome et al. 2021b). Protein quantification was determined using the bicinchoninic acid assay (Pierce BCA Protein Assay Kit, Thermo Fisher Scientific, VIC, Australia). Thirty micrograms of protein lysates were separated by sodium dodecyl sulphate–polyacrylamide gel electrophoresis (SDS-PAGE) using 4–20% Mini-PROTEAN TGX Stain-Free Gels (15 well, Bio-Rad, VIC, Australia). Precision Plus Protein Prestained Standard in All Blue (Bio-Rad, VIC, Australia) was included to determine the molecular weight of bands of interest. Transfer to a PVDF membrane was performed using the semi-dry method (Bio-Rad, Trans-Blot Turbo Transfer System). Incubation with primary antibodies was performed overnight in 5% skim milk in TBST blocking solution at 4 °C on a slow oscillation. Primary/secondary antibodies and related working dilutions are summarised in Table 2. Membranes were incubated with secondary antibody for 1 h at RT before being visualised with chemiluminescence Bio-Rad Clarity Western ECL Blotting Substrate Solution. Images were acquired using the Bio-Rad ChemiDoc MP System (Bio-Rad, VIC, Australia). Densitometry of bands was conducted using Fiji ImageJ, and ratios were normalised to GAPDH, which was used as loading control (Marzagalli et al. 2016).

**Table 2:** List of primary and secondary antibodies used for Western blots

**Antibody**	**Dilution**	**Source (cat. no.)**
Ionised calcium–binding adaptor molecule 1 (Iba1)	1:1000	Abcam (ab133357)
Arginase 1 (Arg1)	1:1000	GeneTex (GTX109242)
Pituitary adenylate cyclase–activating polypeptide (PACAP)	1:1000	GeneTex (GTX37576)
PAC1 receptor	1:1000	GeneTex (GTX30026)
Glyceraldehyde-3-phosphate dehydrogenase (GAPDH)	1:1000	Bio-Rad (VPA00187)
Goat anti-Rabbit IgG HRP	1:10,000	Bio-Rad (STAR208P)

### Statistical Analysis

Statistical analyses were performed using GraphPad Prism version 9.02 for Windows (GraphPad Software, San Diego, CA, USA). All experimental data are reported as mean ± SEM. To assess for statistical differences between two groups (i.e. untreated vs rotenone-treated cells), we utilised the unpaired Student’s *t*-test. Analyses of three or more groups were conducted using one-way ANOVA followed by Sidak’s or Dunnett’s post hoc tests, as appropriate. *P* values ≤ 0.05 were considered statistically significant.

## Results

### Rotenone Induces Polarization in BV2 Microglial Cells

To establish which concentration of rotenone produced sub-toxic but still robust inflammatory effects, to use in subsequent experiments, we determined the effects of increasing concentrations of rotenone (0.0001 to 1 µM) on cell viability and nitric oxide (NO) secretion, an indicator of microglial activation (Contestabile et al. 2012).

**Fig. 2:**
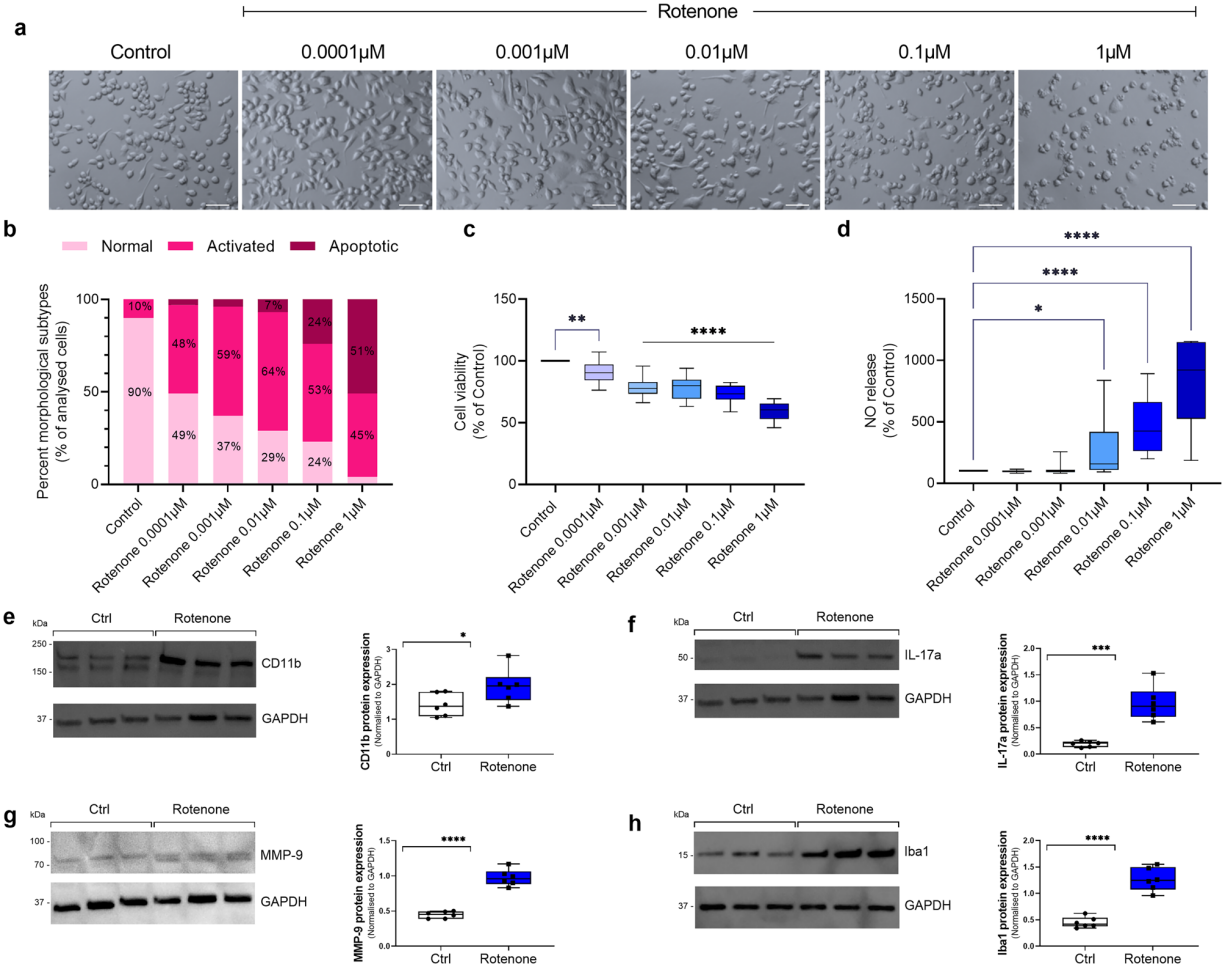
Effects of rotenone on cell viability, nitric oxide (NO) release and expression of microglial activation markers. (**a**) BV2 microglial cells were treated with increasing concentrations of rotenone (0.0001–1 µM) for 24 h, and representative photomicrographs (using embossing filter settings) were taken (scale bar = 60 µm). (**b**) Morphological assessment was performed by assigning cells into three gross categories (normal, activated or apoptotic [please refer to Fig. 1]) and by calculating the percentage of cells in each category. For these analyses, at least eight representative micrographs per condition were appraised (*n* = 8). (**c**) Cell viability and (**d**) NO release were assessed under the same experimental conditions. Representative Western blots and densitometry of (**e**) CD11b, (**f**) IL-17a, (**g**) MMP-9 and (**h**) Iba1 protein expressions in BV2 cells exposed to 0.1 µM rotenone for 24 h. Results are expressed as mean ± SEM. Western blot data represent the mean of two independent experiments; each runs in triplicate (*n* = 6). Bands were normalised to GAPDH, the loading control.*P < 0.05, **P < 0.01, ***P < 0.001, or ****P < 0.0001 vs untreated controls, as determined by one-way ANOVA followed by Dunnett’s post hoc test (**a**–**d**) or unpaired Student *t*-test (**e**–**h**). NO, nitric oxide; IL-17a, interleukin 17a; MMP-9, matrix metalloproteinase-9; Iba1, ionised calcium binding adaptor molecule 1; GAPDH, glyceraldehyde 3-phosphate dehydrogenase

Representative photomicrographs demonstrate the morphological effect of increasing rotenone concentration on BV2 microglial cells (Fig. 2a). Gross morphological analysis revealed a dose-dependent reduction in the percentage of resting cells (from 49% down to 4%) and a steady percentage of activated cells (ranging from 48 to 64%), as identified by flattened and swollen cell somata (Fig. 2b). At the highest concentrations tested (1 µM), rotenone dramatically increased the proportion of apoptotic cells (51%), as opposed to 0.1 µM rotenone, whose percentage was only 24%. These results correlated with a dose-dependent loss of cell viability (*F*_5,87_ = 52.63, ****P < 0.0001) (Fig. 2c).

Accordingly, a dose-dependent increase in NO levels was also seen in rotenone-treated BV2 microglial cells (Fig. 2d). Of note, 0.01 μM was the first dose to record a significant increase in NO compared to untreated controls (*F*_5,78_ = 10.48, *P = 0.0476). Both 0.1 and 1 μM rotenones significantly increased NO levels, compared to untreated controls (****P < 0.0001, respectively) (Fig. 2d).

Based on the above results, we chose 0.1 μM rotenone as the sub-toxic concentration (< 50% apoptotic; Fig. 2a–c) able to trigger significant NO release (Fig. 2d). At this concentration, rotenone also caused significant increase in the protein expression of microglial activation and pro-inflammatory markers, CD11b (*P = 0.0493, Fig. 2e), IL-17a (***P = 0.0002; Fig. 2f), MMP-9 (****P < 0.0001; Fig. 2g) and Iba1 (****P < 0.0001; Fig. 2h).

### PACAP Prevents Rotenone-Induced Nitric Oxide Release but Not Cytotoxicity

**Fig. 3:**
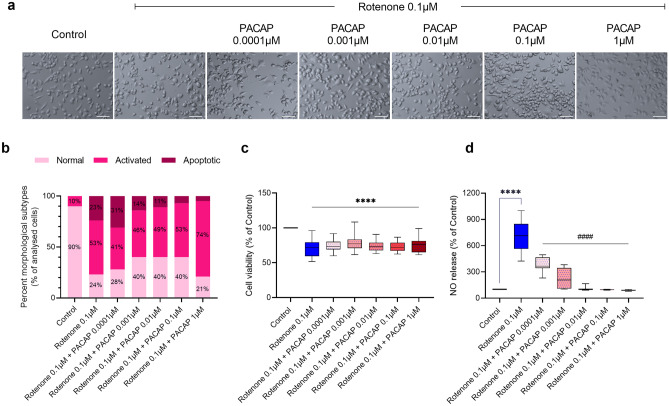
Dose–response effects of PACAP on rotenone-treated microglia. PACAP titration experiments showing dose-dependent changes in morphology, cell viability and nitric oxide release. (**a**) BV2 cells were either treated with rotenone alone (0.1 µM) or co-treated with increasing concentrations of PACAP (0.0001–1 µM) for 24 h and representative photomicrographs (using the embossing filter settings) were taken (scale bar = 60 µm). (**b**) Morphological assessment was performed by assigning cells into three gross categories (normal, activated or apoptotic) and by calculating the percentage of cells in each category. For these analyses, at least eight representative micrographs per condition were appraised (*n* = 8). (**c**) Cell viability and (**d**) NO release were assessed under the same experimental conditions. Data reported as mean ± SEM from at least three independent experiments run using eight biological replicates per group (*n* = 24). ****P < 0.0001 vs untreated controls or ^####^*P* < 0.0001 vs rotenone-treated cells, as determined by one-way ANOVA followed by Sidak’s post hoc test. PACAP, pituitary adenylate cyclase-activating polypeptide; NO, nitric oxide

To determine if PACAP prevents rotenone-induced toxicity, BV2 cells were exposed to 0.1 μM rotenone and treated with increasing concentrations (0.0001 to 1 µM) of the peptide PACAP (Fig. 3).

Results from morphological assessments demonstrated that co-treatment with PACAP dose-dependently reduced the % of apoptotic cells, increased resting/normal appearing cells but had no apparent effects on the subpopulation of cells exhibiting polarised/activated morphology (Fig. 3a, b).

Interestingly, biochemical analyses of cell viability (MTT) revealed that PACAP treatment was not associated with improved viability in BV2 cells at any of the concentrations tested (*F*_6, 105_ = 4.654, ***P = 0.0004; Fig. 3c).

However, in line with the phenotypic changes, we observed a sharp reduction in nitric oxide levels in the supernatant in response to all concentrations of PACAP tested (^####^*P* < 0.0001, compared to rotenone-treated cells; Fig. 3d). Of note, PACAP concentrations of 0.01 μM and above fully prevented nitric oxide release, with levels comparable to untreated controls (*F*_6,101_ = 31.13, ****P < 0.0001; Fig. 3d).

### VIP Prevents Rotenone-Induced Nitric Oxide Release but Not Cytotoxicity

**Fig. 4:**
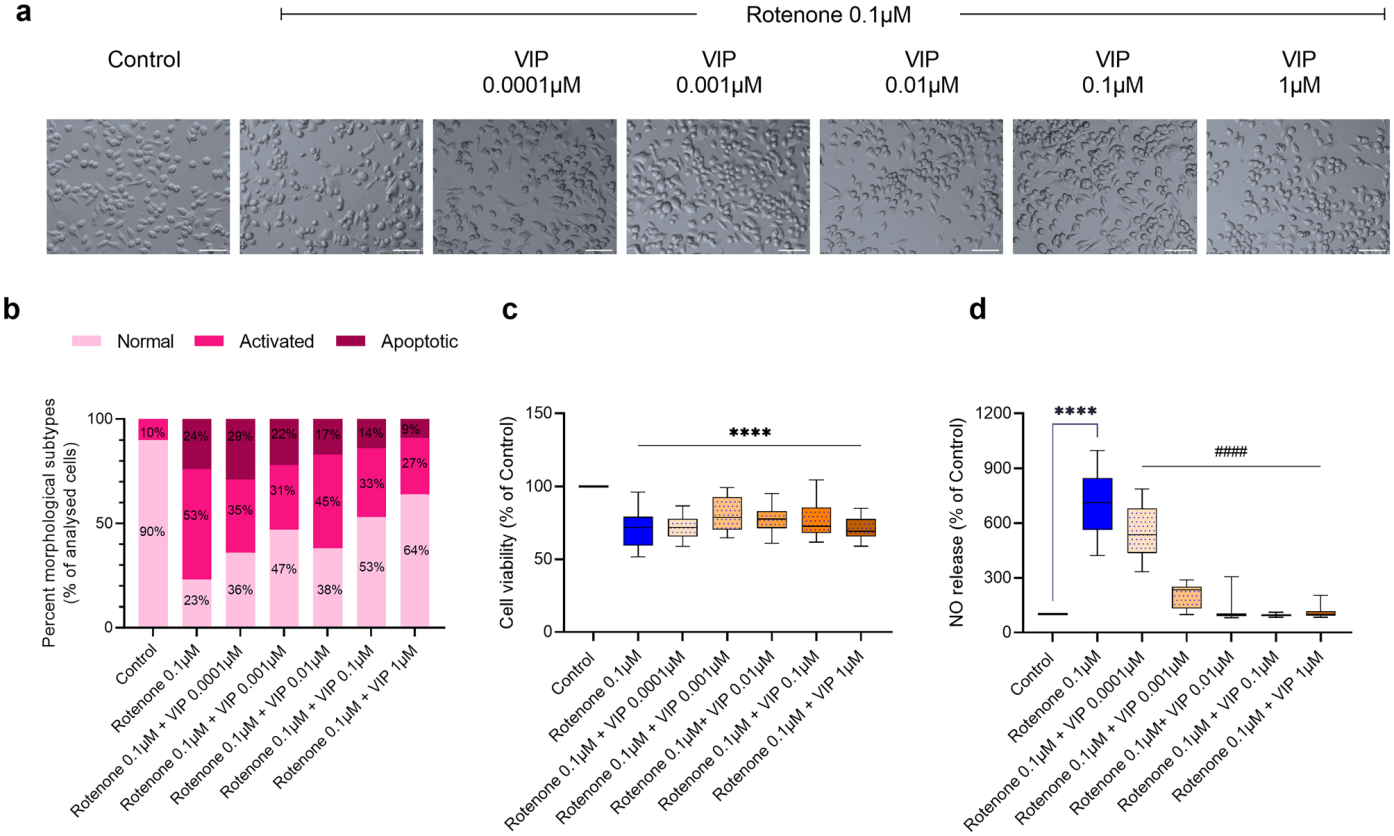
Dose–response effects of VIP on rotenone-treated microglia. VIP titration experiments showing dose-dependent changes in morphology, cell viability and nitric oxide release. (**a**) BV2 cells were either treated with rotenone alone (0.1 µM) or co-treated with increasing concentrations of VIP (0.0001–1 µM) for 24 h and representative photomicrographs (using the embossing filter settings) were taken (scale bar = 60 µm). (**b**) Morphological assessment was performed by assigning cells into three gross categories (normal, activated or apoptotic) and by calculating the percentage of cells in each category. For these analyses, at least eight representative micrographs per condition were appraised (*n* = 8). (**c**) Cell viability and (**d**) NO release were assessed under the same experimental conditions. Data reported as mean ± SEM from at least three independent experiments run using eight biological replicates per group (*n* = 24). ****P < 0.0001 vs untreated controls or ^####^*P* < 0.0001 vs rotenone-treated cells, as determined by one-way ANOVA followed by Sidak’s post hoc test. VIP, vasoactive intestinal peptide; NO, nitric oxide

Similar analyses were performed using increasing concentrations (0.0001 to 1 µM) of VIP on rotenone-treated cells (Fig. 4).

We observed a more obvious anti-inflammatory effect of VIP in comparison with PACAP, as co-treatment with VIP largely prevented the shift of cells towards an activated phenotype and resulted in reduced apoptotic cells (Fig. 4a, b). In contrast, MTT data showed that none of the concentrations of VIP tested reliably prevented rotenone-induced reduction of cell viability (****P < 0.0001, compared to untreated controls; Fig. 4c).

A significant reduction in nitric oxide secretion was observed in response to VIP treatment, at all concentrations (^####^*P* < 0.0001, compared to rotenone treated cells; Fig. 4d). Similarly to PACAP treatment, cells exposed to 0.01 μM and above of VIP reduced nitric oxide release to levels comparable to untreated controls (*F*_6,96_ = 20.30, ****P < 0.0001; Fig. 4d).

Our results align with other studies in similar experimental paradigms, whereby nanomolar concentrations of each peptide were sufficient to produce anti-inflammatory and protective effects (Karunia et al. 2021). Accordingly, we proceeded using 0.01 µM as the preferred peptide concentration for the remainder of this study.

### PACAP and VIP Prevent Rotenone-Induced Microglial Polarization

**Fig. 5:**
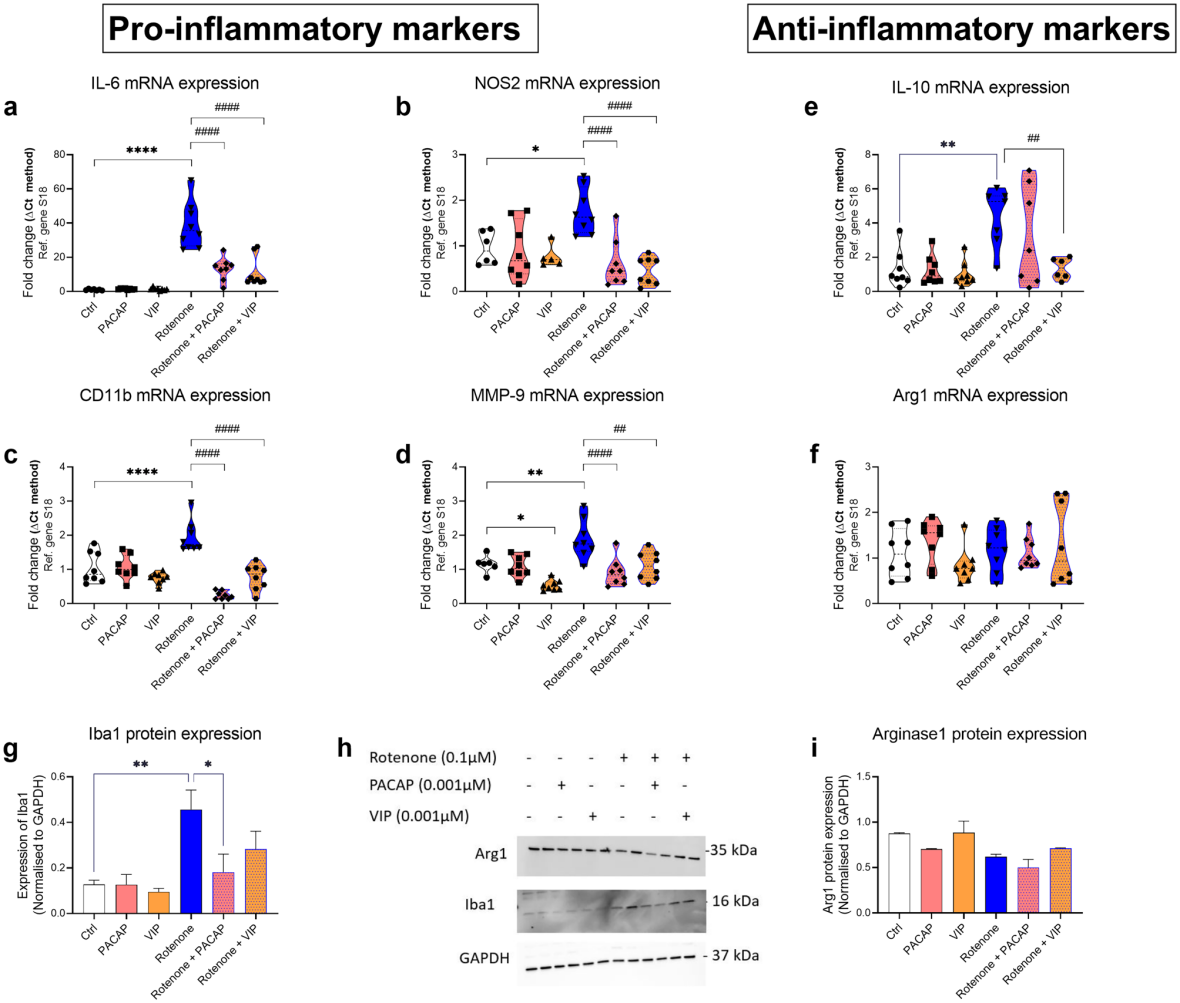
Effects of PACAP or VIP treatment on BV2 microglia inflammatory profile after exposure to rotenone. Real-time qPCR and Western blot analyses of microglial activation markers, pro- and anti-inflammatory cytokines in cells exposed to rotenone alone (0.1 µM), PACAP alone (0.001 µM), VIP alone (0.001 µM) or in combination for 24 h. Gene expression changes of (**a**) IL-6, (**b**) NOS2, (**c**) CD11b, (**d**) MMP-9, (**e**) IL-10 and (**f**) Arg1 in response to treatments are shown. Relative changes in mRNA levels were determined using the ΔCT method and normalised to the ribosomal protein subunit S18, used as the housekeeping gene. (**g**–**i**) Western blot analyses and densitometry of (**g**) Iba1 and (**i**) Arg1 protein expression. Quantifications were performed using the ImageJ software, and band densities were normalised to GAPDH, the loading control. Data represent the mean of 3–6 biological replicates for each group. Results are expressed as mean ± SEM. *P < 0.05, **P < 0.01 or ****P < 0.0001 compared to untreated controls. ^##^*P* < 0.01 or ^####^*P* < 0.0001 compared to rotenone treated cells as determined by one-way ANOVA followed by Sidak’s post hoc test. PACAP, pituitary adenylate cyclase-activating polypeptide; VIP, vasoactive intestinal peptide; s18, ribosomal protein s18; GAPDH, Glyceraldehyde 3-phosphate dehydrogenase; kDa, kilodalton; n.s., not significant; Ctl, untreated controls. IL-6, interleukin-6; NOS2, nitric oxide synthase 2; MMP-9, matrix metallopeptidase 9; IL-10, interleukin-10; Arg1, arginase-1; Iba1, ionised calcium binding adaptor molecule 1

To evaluate if PACAP or VIP co-treatment prevented rotenone-induced microglial polarization, we measured the expression levels of both pro- and anti-inflammatory genes. BV2 cells were exposed to rotenone (0.1 µM) and/or PACAP (0.01 µM) and/or VIP (0.001 µM) for 24 h. As shown in Fig. 5a–d, rotenone treatment strongly increased the gene expression of IL-6 (****P < 0.0001), NOS2 (*P = 0.0120), CD11b (****P < 0.0001) and MMP-9 (**P = 0.0051), compared with untreated controls. Similarly, Iba1 protein expression was increased in response to rotenone (**P = 0.0065). The mRNA expression of the anti-inflammatory cytokine, IL-10, was also significantly increased in response to rotenone treatment (**P = 0.0019; Fig. 5e), but Arg1 levels remained unchanged (*F*_5,42_ = 2.613, *P = 0.0382; Fig. 5f).

Co-treatment with PACAP significantly decreased the expression of IL-6 (^####^*P* < 0.0001; Figs. 5a), NOS2 (^####^*P* < 0.0001; Fig. 5b), CD11b (^####^*P* < 0.0001; Fig. 5c) and MMP-9 (^####^*P* < 0.0001; Fig. 5d), compared to rotenone-treated cells. However, PACAP was unable to prevent IL-10 induction by rotenone (Fig. 5e) and had no effects on Arg1 mRNA expression (Fig. 5f) mRNA expression levels compared to rotenone-treated cells. PACAP was the only peptide to significantly reduce the protein expression of Iba1 in response to rotenone-induced inflammation (^#^*P* = 0.0258) (Fig. 5g, h).

With regard to VIP, peptide co-treatment also reliably decreased the expression of IL-6 (^####^*P* < 0.0001; Fig. 5a), NOS2 (^####^*P* < 0.0001; Fig. 5b), CD11b (^####^*P* < 0.0001; Fig. 5c) and MMP-9 (^##^*P* = 0.011; Fig. 5d) as compared to rotenone-treated cells.

Notably, in contrast to PACAP, VIP co-treatment prevented the induction of IL-10 gene expression caused by rotenone treatment (^##^*P* = 0.0051; Fig. 5e).

### Rotenone Alters the Expression of PACAP, VIP and Related Receptors

**Fig. 6:**
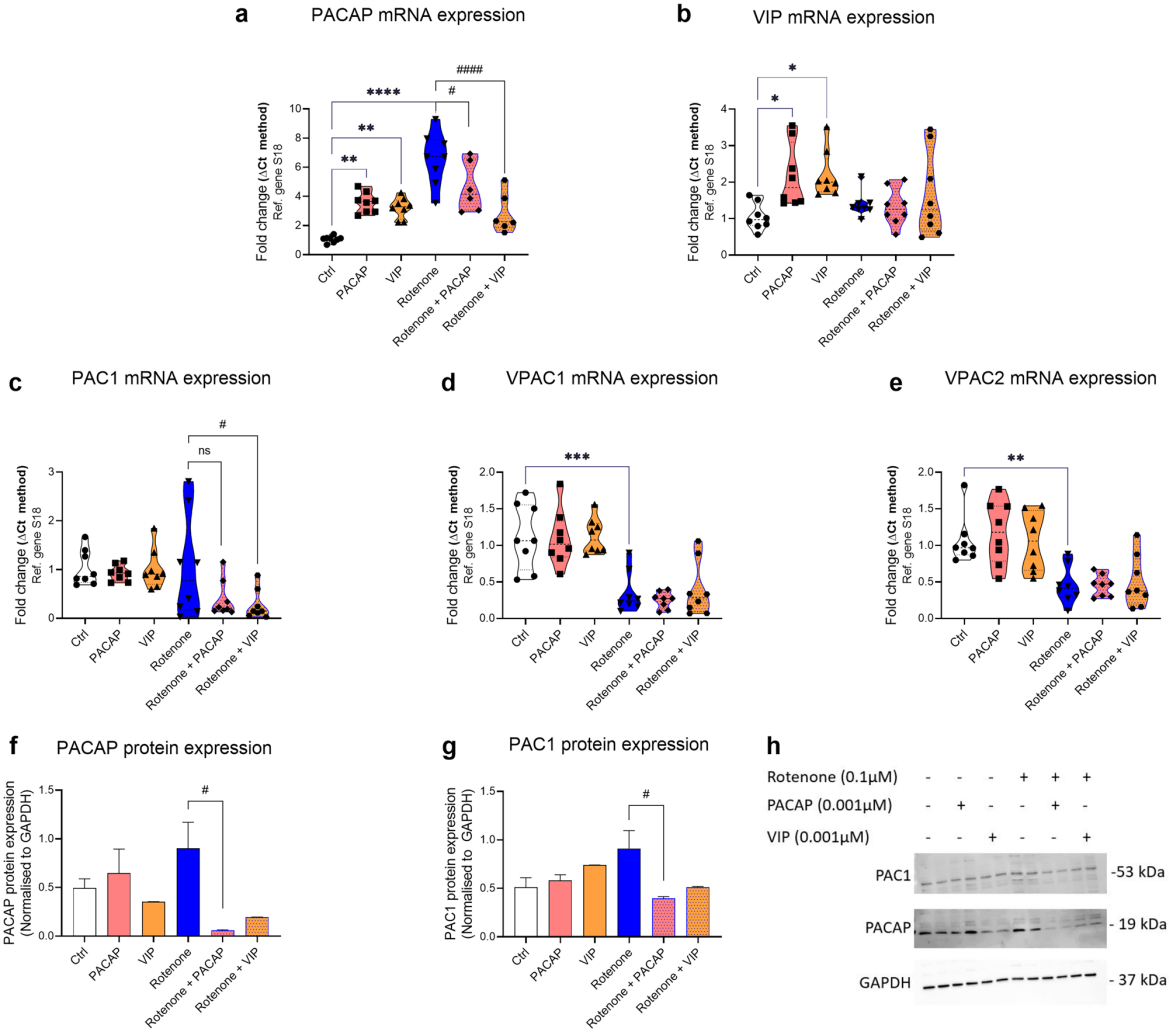
Expression of PACAP, VIP and their receptors in BV2 microglia exposed to rotenone. PACAP, VIP and receptor gene expression after treatment with 0.1 µM rotenone in the presence or not of exogenous PACAP (0.001 µM) or VIP (0.001 µM) for 24 h. Real-time qPCR analyses of (**a**) PACAP, (**b**) VIP, (**c**) PAC1, (**d**) VPAC1 and (**e**) VPAC2 gene expression. Relative changes in mRNA levels were determined using the ΔCT method and normalised to the ribosomal protein subunit S18, here used as the housekeeping gene. (**f**–**h**) Western blot analysis and densitometry of (**f**) PACAP and (**g**) PAC1 protein expression. Quantifications were performed using the ImageJ software, and normalised values were calculated by dividing the mean optical density of bands over the corresponding GAPDH. Data represent the mean of 3–6 biological replicates for each group. Results are expressed as mean ± SEM. *P < 0.05, **P < 0.01, ***P < 0.001 or ****P < 0.0001 compared to untreated controls. ^#^*P* < 0.05 or ^####^*P* < 0.0001 compared to rotenone treated cells as determined by one-way ANOVA followed by Sidak’s post hoc test. PACAP, pituitary adenylate cyclase-activating polypeptide; VIP, vasoactive intestinal peptide; s18, ribosomal protein s18; GAPDH, Glyceraldehyde 3-phosphate dehydrogenase; kDa, kilodalton; n.s., not significant; Ctrl, untreated controls

To determine whether rotenone perturbed the endogenous expression of PACAP, VIP and/or their receptors in BV2 cells challenged with rotenone, real-time qPCR and Western blots were conducted. In the absence of rotenone, PACAP supplementation increased the endogenous mRNA levels of both PACAP and VIP (**P = 0.0014, PACAP gene expression; Fig. 6a; *P = 0.0160, VIP expression; Fig. 6b). Almost overlapping effects were observed with VIP supplementation (**P = 0.0074, PACAP gene expression; Fig. 6a; *P = 0.0121, VIP gene expression; Fig. 6b). However, exogenous stimulation with either peptides did not alter the expression of genes encoding PACAP/VIP receptors (*P* > 0.05; Fig. 6c–e).

Exposure to rotenone significantly up-regulated PACAP transcripts (****P < 0.0001 vs Ctrl; Fig. 6a) and, although not significantly, PAC1 gene expression (Fig. 6c). Rotenone also down-regulated both VPAC1 (***P < 0.001; Fig. 6d) and VPAC2 mRNAs (**P = 0.0038; Fig. 6e).

Co-treatment with PACAP or VIP in cells exposed to rotenone resulted in a significant reduction of PACAP (^#^*P* < 0.05 and ^####^*P* < 0.0001 vs rotenone, respectively; Fig. 6a) but not VIP gene expression (*P* > 0.05 vs rotenone; Fig. 6b). Similarly, PACAP and PAC1 gene expression levels were also reduced by exogenous peptide treatments, although these reductions in gene expression were statistically significant only for VIP (^#^*P* < 0.05) and not for PACAP (*P* = 0.08; Fig. 6c). Neither of rotenone-induced effects on VPAC1 and VPAC2 gene expression were prevented by PACAP or VIP co-treatment (*P* > 0.05 for both genes, respectively; Fig. 6d, e).

To confirm if the preventative activities of PACAP or VIP on rotenone-induced changes in PACAP and PAC1 gene expression could also be seen at the protein level, we performed Western blots. As shown, both PACAP or VIP co-treatments down-regulated PACAP and PAC1 protein expression, although changes were statistically significant in PACAP- (PACAP gene: ^#^*P* = 0.0396 in rotenone + PACAP; PAC1 gene: ^#^P = 0.0321) but not in VIP-cotreated cells  (PACAP gene: *P* = 0.0828 in rotenone + VIP; PAC1 gene: *P* = 0.0938 in rotenone + VIP; Fig. 6f–h).

## Discussion

In this study, we tested whether rotenone, a well-known PD-mimetic, was capable of inducing microglial polarization and appraised whether PACAP or VIP co-treatment could prevent it. For this purpose, we utilised murine BV2 microglial cells, as these cells share several biological and biochemical properties with primary microglial cultures (Anja Henn 2009). Our data indicates that rotenone reduces viability and causes microglial polarization of BV2 microglial cells. Interestingly, neither peptide was able to prevent cell death caused by rotenone toxicity; however, both prevented microglial activation, expression of pro-inflammatory factors and release of nitric oxide. Additionally, we demonstrated that rotenone treatment perturbed the endogenous gene expression levels of both peptides and their related receptors, and this was partly prevented by PACAP or VIP supplementation.

The progressive degeneration of dopaminergic neurons and inflammatory processes are closely related in PD (Hunter et al. 2007; Jing et al. 2021) It has been shown that LPS-induced inflammation promotes the degeneration of dopaminergic neurons, which was not reversed at 21 days post-lesion (Castano et al. 1998). Rotenone has become a popular mimetic utilised to induce PD-like pathology, including dopaminergic degeneration and neuroinflammation in several pre-clinical models of PD (Cannon et al. 2009; Dawson et al. 2018; Gamber 2016). Furthermore, studies have determined that reduction in microglial activation is crucial in promoting neuroprotection in rotenone models of PD (Jayaraj et al. 2021; Jing et al. 2021). Along with the evidence of microglial activation in *post-mortem* PD brains (Badanjak et al. 2021), these studies suggest that microglia polarization and the consequent accumulation of pro-inflammatory factors may be critical in promoting PD pathogenesis. Vice versa, inhibition of microglia activities has been indicated as a viable strategy to attenuate neurodegeneration, relieve motor symptoms and slow disease progression (Gupta et al. 2018).

In animal studies, recent evidence has shown that rotenone-induced microglial activation precedes neurodegeneration (Zhang et al. 2021). Therefore, the identification of treatments that can inhibit inflammation represents an attractive strategy to prevent the ongoing neurodegeneration seen in PD and other neurodegenerative conditions.

In line with other studies, we demonstrated that rotenone induced the expression of the microglial activation and the pro-inflammatory markers Iba1 and IL-1β in BV2 microglial cells (Li et al. 2021; Zhang et al. 2020). We also report that both PACAP and VIP are able to mitigate rotenone-induced inflammation and microglial activation in BV2 cells. These observations align with our previous data indicating that these peptides exert immunosuppressive effects in LPS-treated BV2 cells (Karunia et al. 2021). Furthermore, it corroborates previous evidence demonstrating that PACAP provides protection against MPP + neurotoxicity to primary rat mesencephalic neuron-glia cultured only in the presence of microglia, suggesting that an essential component of PACAP neuroprotective function is achieved via microglial inactivation (Yang et al. 2006). This is strengthened by studies demonstrating neuroprotective effects of PACAP administration against rotenone toxicity (Maasz et al. 2017; Wang et al. 2005). Similarly to PACAP, the neuroprotective effect of VIP has also been associated with its ability to block/prevent microglial activation and reduce the expression of pro-inflammatory mediators in a MPTP mouse model of PD (Delgado and Ganea 2003). Some studies have enhanced the activity of VIP through TAT-tagging, which allowed for positive allosteric modulation of the PAC1 receptor, resulting in a more protective effect against MPTP neurotoxicity than PACAP alone (Yu et al. 2020). The present findings further strengthen such in vivo evidence in a controlled monoculture, thereby excluding the potential influence of other glial cells.

Previous reports have demonstrated that PACAP and VIP reduced the inhibition of cytokine production by lipopolysaccharide in primary microglia (Delgado et al. 2002), an effect that was mediated by the VPAC1 receptor. In contrast, our results pinpoint the PAC1 receptor as the main player of PACAP and VIP modulatory activities in BV2 microglial cells exposed to rotenone. This is not surprising, as the nature of the insult causing inflammation in the two studies is different. In addition, despite the similarities with primary microglia, we cannot exclude that some differences might exist in the way BV2 vs primary microglia regulate the expression of this class of receptors in response to a toxic stimulus. Mechanistic gain and loss-of-function studies are warranted to dissect the specific contribution of PACAP/VIP receptors in regulating inflammatory responses. However, it should be noted that studies in PAC1 receptor knockout mice proposed that the PACAP/PAC1 axis is at the forefront of the anti-inflammatory response seen in mice (Martinez et al. 2002; Zeng et al. 1998), supporting the notion that the expression and activity of the PACAP/VIP protective/anti-inflammatory system may be influenced by several factors, including the response of neighbouring glia or neurons to an inflammatory microenvironment. In fact, microglia are in constant communication with astrocytes to regulate the immune responses within the CNS (Morales et al. 2021), and both PACAP and VIP play an important role in mediating the activity of astrocytes (Masmoudi-Kouki et al. 2007). This is further validated in a recent study that demonstrated increased axonal pathology and expression of microglia activation markers following the conditional deletion of the PAC1 receptor in retinal neurons (Van et al. 2021).

In conclusion, our study has demonstrated that stimulation with PACAP or VIP reliably prevented the induction of NO release, microglial activation markers and pro-inflammatory cytokines in BV2 microglia exposed to rotenone. In addition, our findings reveal that the toxicant perturbs the endogenous expression of PACAP/VIP peptides and receptors in a way that differs from that seen with other inflammatory mimetics. This raises the possibility that upon rotenone exposure, microglial cells need to re-adjust the expression of peptides and receptors to enable the activation of both protective and anti-inflammatory pathways, rendering cells able to cope with the detrimental effects of this toxicant. Nonetheless, whilst no in vitro model can recapitulate all the pathogenic features of PD, these results bring us a step closer into our understanding of the potent immune modulatory role elicited by these peptides and recommend their consideration as potential targets to relieve the chronic inflammation and microglial activation observed in several neurodegenerative disorders of the CNS, where an inflammatory component is present.

## Data Availability

All data generated or analysed during this study are included in this published article. Also, labelled original/full blots of all the Western blots included in this study can be made available upon reasonable request to the authors.

## Abbreviations

PD: Parkinson’s disease
CNS: Central nervous system
PACAP: Pituitary adenylate cyclase-activating peptide
VIP: Vasoactive intestinal peptide
LPS: Lipopolysaccharide
NO: Nitric oxide
IL-17a: Interleukin-17a
MMP-9: Matrix metallopeptidase 9
Iba1: Ionised calcium-binding adapter molecule 1
IL-6: Interleukin-6
NOS2: Nitric oxide synthase 2
IL-10: Interleukin-10
Arg1: Arginase-1
